# What Do Children Think of Their Perceived and Ideal Bodies? Understandings of Body Image at Early Ages: A Mixed Study

**DOI:** 10.3390/ijerph18094871

**Published:** 2021-05-03

**Authors:** María Pilar León, Irene González-Martí, Onofre Ricardo Contreras-Jordán

**Affiliations:** Department of Physical Education, Arts Education and Music, University of Castilla-La Mancha, 02071 Albacete, Spain; Irene.GMarti@uclm.es (I.G.-M.); Onofre.CJordan@uclm.es (O.R.C.-J.)

**Keywords:** preschoolers, self-perception, body satisfaction, body image

## Abstract

Research into children’s body perceptions and ideals is scarce despite evidence of body dissatisfaction in childhood. This study aimed to understand preschoolers’ body image by employing a mixed design. Using a novel figural scale (Preschoolers’ Body Scale) that comprises four child figures ranging in BMI, 395 children ages 4–6 (54% boys) selected their perceived and ideal body and explained why they picked these bodies. Children tended to underestimate their body size and many of them desired slimmer bodies, especially girls and older participants, although body-size perception improved with age. Most children showed body satisfaction, especially boys and younger children. Ideal body choices were not always explained by beauty ideals but by physical abilities, desire to grow up, mothers’ comments, and nutrition. Many responses reflected limited body awareness, suggesting body image may not yet be fully formed in preschoolers due to their incipient cognitive development.

## 1. Introduction

Young populations have been the target for some authors who have aimed to know the roots of body image development given the presence of eating disorders in childhood [1]. Body image, understood as the perception of one’s own body and the feelings and behaviors associated with such perception, has been investigated with children, mainly focusing on the perceptual and cognitive-affective components, typically using visual methods such as figure-rating scales [2]. Studies investigating the perceptual component of body image have concurred that preschoolers are not able to accurately report their body size [3,4,5,6]. This suggests that greater accuracy in the estimation of body size develops later [7], as before the age of five, children have difficulties judging their body size and do not have a reliable mental representation of their body mass index (BMI) [8].

The inaccuracy in body size perception seems to tend towards underestimation, especially as children become older [5,9]. However, Gardner [10] and Ra et al. [6] stated that young children commonly overestimate their body size. It is not clear why children select a certain figure as perceived and why the high prevalence of inaccurate perception is observed. Xu and Nerren [11] considered that the main reason is insufficient cognitive development. This inaccuracy can also be explained by rudimentary body awareness, which is the basis of body image [12], or by a bias towards obesity. Children may already be aware of societal appearance ideals and the stigmatisation towards obesity, which could affect their perceptions [3,13].

The cognitive-affective component of body image has also been measured among children. Literature illustrates that weight concerns and body dissatisfaction can emerge at very early ages [14]. However, the age at which such dissatisfaction becomes a cause of concern for the future development of physical and mental health problems remains unclear [15]. Evidence suggests that more than half of children aged between 3 and 6 years are dissatisfied with their body and want to be thinner [16,17,18], although Smolak [14] stated that is not until about 6 years when children experience body dissatisfaction. Some authors suggest that discontent may occur because preschoolers have already formed attitudes about the body and show discrimination towards obese bodies [3,19]. Alternatively, body dissatisfaction could be explained by the engagement in social comparison and the adoption of societal standards of appearance at a very early age [14]. In Western society, these standards of beauty are based on muscular and lean bodies for males and thin bodies for females [14].

Among preschoolers, girls are more likely than boys to have body dissatisfaction, expressed as the discrepancy between perceived and ideal body size [9]. This appears to increase as children become older [8,16,18]. According to Grogan [20], by the age of 8, body-related concerns are similar to those of adults. However, other studies claim that most preschoolers are satisfied with their bodies [17,21].

Thus, because findings from research based on quantitative data are mixed and unclear, research that incorporates qualitative data would help to further examine and understand body image among preschoolers and identify the reasons that lead them to select a certain body as perceived or ideal [14]. While qualitative research on this topic is thus far scarce among preschoolers, studies using interviews have found that young children were more likely to express concern about their clothes or hair than about their body, although sometimes boys showed concern for muscles and girls for body weight [17,22]. Furthermore, it has been observed that physical abilities and fitness are important for very young children [13] and preadolescents [23] when it comes to judging their body image. Qualitative data have also shown the influence of the social context on older children’s body image: for example, the media and peers [23] or mothers’ comments about their daughters’ bodies [24]. However, less is known about what factors preschoolers consider important when they select their perceived and ideal body.

The purpose of this research was to know the preschoolers’ thoughts about their perceived and ideal body in order to acquire a greater understanding of their body image. A mixed design was employed to measure body size perception, body size ideal, and body satisfaction. According to this aim, and based on the discussed literature, the following hypotheses were established: (1) about half of the children would have an inaccurate perception of their body size, and this perception would improve over the years but was not expected to differ by gender; (2) girls and older children will have a slimmer body ideal; (3) children will be generally satisfied with their bodies, especially boys and younger children; and (4) children’s motives to select their perceived and ideal body will be based on several aspects that they consider important, which would not be always related to body size or shape.

## 2. Materials and Methods

### 2.1. Participants

The convenience sample comprised 395 preschoolers, 213 boys and 182 girls, aged 4 to 6.4 years (*M* = 5.11 ± 0.64), recruited from 13 schools in Albacete, Spain. According to ethnicity, 98.4% were Caucasian, 0.7% Amerindian-European, 0.5% Black, 0.2% Asian, and 0.2% Black-European. The socioeconomic level of the families was medium-to-high. Most parents had higher education (41.3%), and their combined monthly household income was between €2000 and €2500 in 20% of cases. To analyse the data, a stratification by age was made given the possible cognitive differences between children born in different months of the same calendar year. Thus, children were divided into five groups: 4 to 4.4 years (*n*♀ = 27; *n*♂ = 51), 4.5 to 4.9 years (*n*♀ = 38; *n*♂ = 55), 5 to 5.4 years (*n*♀ = 39; *n*♂ = 40), 5.5 to 5.9 years (*n*♀ = 53; *n*♂ = 46), and 6 to 6.4 years (*n*♀ = 25; *n*♂ = 21). It is important to note that those children with special educational needs were not included in this study unless their special needs did not bias the results (e.g., one child with a severe visual impairment did not participate in the study because this participant was not able to see clearly the figural stimuli).

### 2.2. Instruments and Measures

#### 2.2.1. Preschoolers’ Body Scale (PBS)

The scale [25] is composed of eight grayscale body figures belonging to four boys and girls and has demonstrated good validity and reliability for being used with preschoolers. The scale was developed from photographs of children aged 4 to 6 years old. The figures are arranged from the thinnest to the heaviest and shown in front and profile views. Each figure represents a different BMI, BMI percentile, and weight status. The first figure represents a very low-weight child, 3rd percentile (BMI♂ = 13.13 and BMI♀ = 13.03); the second figure represents a normal weight child, 50th percentile (BMI♂ = 16 and BMI♀ = 15.06); the third figure represents an overweight child, 85th percentile (BMI♂ = 17.1 and BMI♀ = 17.06); and the last figure represents an obese child with >99th percentile (BMI♂ = 21.03 and BMI♀ = 21.25).

This figure-rating scale was used as visual instruments are considered the most suitable and common for preschoolers [2]. Other figure-rating scales [26] have been used in previous studies with preschoolers. However, these have not been demonstrated to be valid and reliable for such early ages.

To measure body-size perception, body ideal, and body satisfaction, children were presented with the eight figures from the PBS of the same gender as the participant, four from a profile view and four from a front view.

Based on literature [2], we established two closed-ended questions to know the child’s perceived and ideal figure: “Which child looks most like you?”, “Which child would you most want to look like?”. Furthermore, as Birbeck and Drummond [13,27] did, we included two open-ended questions to qualitatively assess why children chose a certain figure as perceived or ideal: “Why do you think this boy/girl looks like you?”, “Why do you want to look like this boy/girl?”

Following previous studies [9,21], body-size perception and body satisfaction were calculated using two discrepancy scores. The first score was obtained by subtracting the BMI of the perceived figure from the child’s actual BMI. A negative score meant that the children perceived themselves to be thinner than their real size (i.e., underestimate their body size), whereas a positive score meant that the children overestimated their real body size. For body satisfaction, the BMI of the ideal figure was subtracted from the BMI of the perceived figure. A negative score reflected the desire for thinner bodies than theirs, and a positive score revealed the desire to be larger or obese.

#### 2.2.2. Participant BMI and BMI Percentiles

Children were measured to calculate their BMI and BMI percentiles. Their height and weight were measured with light clothing and without shoes, using a portable stadiometer accurate to 0.1 cm (Tanita HR-001) and a digital scale (Tanita HD-366) accurate to 0.1 kg. These measurements were taken by a specialised researcher with the first-level anthropometry certification from the International Society for the Advancement of Kinanthropometry (ISAK).

### 2.3. Procedure

To develop the study, we first received the approval of teachers and school principals. Parents, after reading the purpose and procedure of the study, provided their written consent and children provided their verbal consent prior to data collection, which was conducted during school time.

The children’s anthropometrics were obtained individually and following the ISAK protocol [28]. Next, each child was interviewed for approximately 15 min by the first author. The same protocol was applied with each participant, and a confident environment was created to foster dialogue. Before conducting the test, the task was explained to the children: the purpose was to collect their opinions, and thus there were no right or wrong answers. Figure scales were displayed on a 17-inch screen: first, the profile figure (PF) scale and then the front figure (FF) scale.

Depending on the children’s responses concerning the reason for choosing their perceived and ideal figure, new questions were asked to obtain a greater knowledge of their body image and clarify the concepts used to define their perceptions, as Delval [29] recommends. One example of these added questions is the following: —child: “I want to look like this boy because he is cool”—interviewer: “Why do you think this boy is cool?”. Delval [29] also points out that children use language in a manner that does not always coincide with that of adults. Therefore, the interviewer did not suggest response options to the children and did not assume the meaning of children’s responses unless it was completely clear. Also, if the child provided incomplete or meaningless answers, there was little emphasis on repeating the questions and extracting more information because it could increase the likelihood of not-honest answers [27].

Before children selected the ideal figure, they were told that they could choose the same figure for perceived and ideal. As Tatangelo et al. [15] pointed out, some works have not indicated whether children were given the option of selecting the same figure as perceived and ideal; thus, they might think that for each question they should select a different figure. To ease the task and follow the recommendation of Hill [2], before asking the children about their body ideal, they were reminded of the figure they selected as perceived.

The child’s responses were recorded and subsequently transcribed. Some children did not respond to all of the questions.

This study was approved by the Hospital Research Ethics Committee of Albacete [Acta nº 09/2017].

### 2.4. Analysis

#### 2.4.1. Quantitative Analysis

Children’s anthropometrics were calculated using AnthroPlus 1.0.4 (World Health Organization, WHO) [30]. Data were analysed using SPSS 24.0 (IBM, Armonk, NY, USA). Descriptive statistics were calculated for the sample demographics and main variables. Spearman’s correlations and mean differences were conducted through the Kruskal–Wallis and Mann–Whitney U tests due to the non-normal distribution of data and the nature of the variables.

#### 2.4.2. Qualitative Analysis

Children’s responses to the open questions were transferred to Atlas.ti 8.4.15 (Cleverbridge AG, Köln, Germany) for coding. The information was analysed following a thematic analysis approach, which involves the identification of recurring themes as well as the description and interpretation of several aspects related to the research topic [31].

After familiarisation with the transcribed material, the three authors separately noted relevant concepts and created preliminary codes. Several meetings were arranged to group common codes and resolve discrepancies. Once the final codes were created, the first author briefly described each of them, which was verified by the other authors. Subsequently, codes were grouped into themes and subthemes by each of the authors and discussed in a meeting to establish the final list.

The first author coded the entire data set, and the other two authors identified the categories and subcategories in 30% of the children’s responses to understand the inter-rater reliability. Fleiss’ Kappa was calculated, and we obtained K = 0.93. Finally, we held meetings to discuss some responses and noticed that data saturation had occurred.

## 3. Results

### 3.1. Anthropometry

Following the WHO standards [32], 11.1% of the children were low weight (percentile ≤10), 67.3% were normal weight (percentiles 15–75), 14.2% were overweight (percentiles 85–95), and 7.4% were obese with a percentile ≥97. According to gender, 14.1% of boys (*n* = 30) and 7.7% of girls (*n* = 14) were low weight; 64.8% and 69.8%, respectively (*n*♂ = 138 y *n*♀ = 127), were normal weight; 13.6% of boys (*n* = 29) and 15.4% of girls were overweight (*n* = 28); and 7.5% of boys (*n* = 16) and 7.1% of girls (*n* = 13) were obese.

### 3.2. Quantitative Data

#### 3.2.1. Body-Size Perception

As shown in Table 1, 20.4% and 32.2% of children accurately perceived their body size using PF and FF, respectively. We observed an improvement, although not linear, in the accuracy of body size perception with FF, and this improvement was more noticeable after 5 years of age, where success rates ranged from 30.4% to 41%.

The perception was influenced by age, obtaining significant differences with PF, χ^2^ (4, *N* = 388) = 13.39, *p* = 0.01, as children tended to underestimate their body size more as they became older, with percentages higher than 70% and with average discrepancy scores of −0.82 with PF and −0.11 with FF (Table 2).

Compared with boys, girls tended to perceive themselves as thinner, although no significant differences were observed (PF: *z* = −0.22, *p* = 0.82; FF: *z* = −1.67, *p* = 0.93).

#### 3.2.2. Body Ideal and Body Dissatisfaction

Table 1 shows that 34.8% of children with PF and 30.2% with FF wanted a different body. These levels reveal that many children wanted to have the same body as their perceived one, which is also shown in Table 2, where the average discrepancy score (ideal BMI minus perceived BMI) is close to 0 with FF (*M* = −0.14 ± 2.1) and PF (*M* = 0.36 ± 2.58). In all age groups, males reported higher satisfaction (i.e., children chose the same figure as perceived and ideal) than females, with percentages between 70% and 80.9%. The prevalence of body satisfaction was similar in all ages, and significant differences were not observed in either PF χ^2^ (4, *N* = 388) = 3.97, *p* > 0.05, or FF χ^2^ (4, *N* = 394) = 6.7, *p* > 0.05.

The desire for thinner bodies increased with age in both genders, with PF χ^2^ (4, *N* = 381) = 21, *p* < 0.001, and FF χ^2^ (4, *N* = 391) = 15.27, *p* < 0.005, and was higher among females with both scales (range between 11.5% and 36% females vs. 5% and 14.3% males). Additionally, a notable difference by age was observed in females: an increased desire from 18.5% at 4 years to 36% at 6 years. Conversely, the desire for larger bodies decreased substantially from 5.5 years of age in both genders. Indeed, at 6 years of age, 4.8% and 4% of boys and girls with FF wanted a larger body than their perceived body, although these percentages were much higher at all other ages.

The Kruskal–Wallis test demonstrated that body ideals were significantly different among age groups based on gender; in the case of girls, this was observed both with PF χ^2^ (4, *N* = 174) = 17.34, *p* = 0.002 and FF χ^2^ (4, *N* = 182) = 17.92, *p* < 0.001, and in males, the difference was only observed with PF (χ^2^ [4, *N* = 207] = 10.59, *p* < 0.031).

### 3.3. Qualitative Data

Six main themes emerged when children were asked to select their perceived and ideal bodies. See Table 3 for descriptions and details of each theme.

Theme 1: Body size perception. The children’s judgements were not always based on the body size or its parts but also on body attributes or other attributes that did not provide information on body size or shape.

Body size. One-third of the children showed awareness of body size when they were asked why they selected a particular figure as representing their perceived body; for example, “because she’s medium and I’m medium” (girl, 5.7 years, actual BMI = 17.7, selected BMI = 15.06); “because I’m very skinny” (boy, 6.2 years, actual BMI = 17.12, selected BMI = 13.13); “because she is a bit chubby like me” (girl, 5.5 years, actual BMI = 14.54, selected BMI = 17.06); and “because she has the same body than me” (girl, 5.3 years old, actual BMI = 12.77, selected BMI = 15.06).

The reference to terms of thinness, obesity, medium-size, or body size in general notably increased with age, occurring in 15.4% of cases of children from 4 to 4.4 years, whereas the percentage was higher than 36% from 5 years old. These terms were used by both genders in a similar way.

Weight-related aspects of their bodies. Some children focused on body parts where weight or fat are more noticeable, such as the belly, legs, and arms (e.g., “She has a chubby tummy”—girl, 5.6 years old, BMI = 15.95; “He has slimmer legs”—boy, 6.2 years old, BMI = 18.15; “She has slimmer arms”—girl, 5.1 years old, BMI = 16.96). The belly was the body part most mentioned by children, followed by the legs. Compared with males, females referred more times to the size of certain body-related aspects. Regarding age, older children mentioned more body parts that provided information on body size.

Non-weight-related physical aspects of children’s bodies and other aspects. Many children (*n* = 102) focused on body parts that objectively provided little or no information on body size, such as neck, navel, hands, feet, shoulders, and nails (e.g., “because I have a navel”—girl, 4.3 years old, BMI = 15.67; “because he has his feet like mine”—boy, 5.8 years old, BMI = 13.97; “because she has her hands like mine”—girl, 6 years old, BMI = 14.7). We observed that many children mentioned isolated body parts instead of contemplating the body as a whole in terms of size or shape.

Apart from body-related aspects, other attributes were observed such as clothes (e.g., “I have shorts”—girl, 4.3 years old, BMI = 15.67), and the age (e.g., “because he’s the same age”—boy, 6.2 years old, BMI = 14.82; “because she’s my age”—girl, 4.7 years old, BMI = 21). Children aged 6 years provided the least answers in this subtheme. Regarding gender, no notable differences were observed.

Theme 2: Conceptual confusion of dimensions. Forty children revealed conceptual confusion among several dimensions (e.g., tall, short, large, small, thin, obese), especially the younger children, because as the children’s age increased, their confusion about the dimensions decreased. This conceptual confusion was higher among females than males.

Children sometimes used the words big or small to allude to the height: several short children perceived themselves to be smaller, or those who were taller perceived themselves as larger. For example, a 5.8-year-old girl (BMI = 16.02) chose Figure 1 (BMI = 13.03) as the perceived body saying, “because I’m small, I’m short”. The terms big and small were also associated with body weight; e.g., a boy, 5.3 years old (BMI = 13.6) explained, “I would like to look like this, the fat one (pointing to Figure 4, BMI = 21.03), because I like it, because I want to be large like him”. Size was also associated with age: a 5.1-year-old boy (BMI = 13.67) claimed: “I’m very large, I’m super tall, the tallest of my classmates. I’m already 5 years and then they are smaller”.

Some children associated height with thinness and others with obesity, despite all figures having the same height (e.g., girl, 6.3 years old, BMI = 15.72: “I want to look like this because she is very tall”—chose Figure 1, BMI = 13.03; boy, 4.05 years old, BMI 12.2: “He looks like me because he’s very tall”—chose Figure 4, BMI = 21.03).

Theme 3: Influence of the social context. This theme included social aspects that influenced children’s body-size perception and body ideals, such as weight stigmatisation, beauty ideals, and nutrition.

Weight stigmatisation. Although few cases of weight stigmatisation were observed (*n* = 5, of which 4 were boys), the data suggest that discrimination towards obesity is present from early ages. A 5.3-year-old boy (BMI = 15.64) who chose Figure 1 (BMI = 13.13) as perceived body said, “because I don’t have a fat tummy. I see a potbelly and I crack up”. This bias against obesity is also visible in the choice of the desired figures, for example, boy (4.4 years, BMI = 17.15): “I would like to look like this boy (points to Figure 2, BMI = 16) because he’s thin and cooler...Chubby children aren’t cool because they eat a lot of things and get angry, although the thin ones also get angry”.

Beauty ideal. Four children reflected mainstream beauty ideals and the association between thinness and beauty. A girl aged 6.1 years (BMI = 14.64) stated that she wanted to be thinner to be prettier. Responses related to beauty ideals were more predominant with an increase in age: at the age of 6 years, in 4.3% of cases and for the rest of the ages, the percentages were less than 1.3%. These beauty ideals were more frequent among girls (1.65% vs. 0.47%); that is, fewer comments on the muscular beauty ideal in males were observed.

Nutrition. Fifteen children, especially males, demonstrated awareness of the relation between the amount or type of food (i.e., healthy or unhealthy) and specific body size or shape, as exemplified in the following answers. To the interviewer’s question of “Why do you think this boy looks like you?”, a 5.7-year-old boy (BMI = 18.9), after selecting Figure 1 (BMI = 13.13), replied: “because I eat salad and I’m as thin as him”. To this same question and after choosing Figure 1 (BMI = 13.13) as the perceived body, a 4.3-year-old boy (BMI = 13.4) replied: “because I eat little; I’m slim”. Conversely, a 4.5-year-old boy (BMI = 16.36) chose Figure 3 (BMI = 17.1) and expressed: “because I’m a little chubby because I eat a lot”.

Some food-related responses reflected that body-size perception was sometimes obtained from concrete experiences that had recently occurred. Some children perceived themselves as obese because they ate a lot the day before, regardless of their actual weight. For example, a 4.4-year-old boy (BMI = 13.95) who chose Figure 1 (BMI = 13.13) shared:

- Interviewer: “Why do you think this boy looks like you?”
- Boy: “Because he’s a bit fat”.
- Interviewer: “Do you think you are fat?”
- Boy: “Yes, a little because yesterday I ate a lot; I ate a lot of chicken wings”.

Regarding the ideal body, a girl aged 6.1 years (BMI = 25.57) wanted a slimmer figure and to have a smaller tummy. When the interviewer asked her why she wanted to have a smaller tummy, the girl explained: “because my mum says that it is necessary to do sport and not to eat candies nor much chocolate”.

Theme 4: Mother’s influence. Some responses reflect the prominent role of families in the development of body image and the transmission of beauty ideals. In this study, all the children’s comments referred to their mothers except for one case: a grandmother was mentioned by her 5.5-year-old granddaughter (BMI = 14.54) who perceived herself as chubby because her grandmother told her that.

Mother’s influence was illustrated by 13 participants, especially the females: 6.04% of girls and 0.94% of boys mentioned their mothers. Regarding perceived figure, a 5.7-year-old girl (BMI = 15.3) selected Figure 1 (BMI = 13.03) and explained: “because I’ve lost weight; my mother tells me that”. Similarly, a 4.5-year-old girl (BMI = 14.78) commented: “My mother always tells me that I’m very skinny”. Another example was the response of a 6.1-year-old boy (BMI = 14.15):

- Boy: “My mother wants me to be slimmer”.
- Interviewer: “Does she tell you that?”
- Boy: “Yes, because that’s how I’m cuter”.
- Interviewer: “And what does she tell you?”
- Boy: “That I’m good-looking being slim, but I don’t like at all being chubby”.

As for the desired figure, comments from the mothers were also observed. For instance, a 5.9-year-old girl (BMI = 21.25), after choosing a desired figure thinner than the perceived one, added: “I don’t like to be very fat because my mum tells me that I’m too fat”. Another example is that of a 6.3-year-old girl (BMI = 16.02) who said she wanted to be more obese: “because my mum tells me I’m thin like an asparagus”.

The role of mothers as a model for children can also be reflected in some responses related to pregnancy. For some girls, a large belly represented pregnancy, and they chose figures 3 or 4 because they would like to be mums; others chose the thinnest figures because they did not want to look pregnant. When the researcher asked: “Why do you want to look like this one?” a girl aged 4.3 (BMI = 14.59) replied: “because she has a little bigger belly like mums”. Conversely, a 5.6-year-old girl (BMI = 13.83) stated she wanted to be slimmer because Figure 3 (BMI = 17.06) was too fat. To obtain more information from her, the interviewer asked: “So you don’t like being fat?” and the girl explained: “No, because it seems that I’m fat and I’m going to have a baby and I don’t like having children”. Other children, such as a 4.9-year-old child (BMI = 17.05), selected obese figures because their mothers were or had recently been pregnant.

Theme 5: Physical abilities. The children, especially the males (3.75% vs. 1.1% females), valued the functionality of the body. They associated an obese body with musculature and strength whereas thin bodies were associated with greater endurance and speed compared with obese bodies. A 4.7-year-old boy (BMI = 15.3) chose Figure 4 (BMI = 21.03) as the perceived body and stated: “because I’m strong”, and a 5.7-year-old boy (BMI = 18.9) chose Figure 1 (BMI = 13.13) and stated: “because I run very fast”.

Regarding the ideal body, a 4.5-year-old boy (BMI = 14.5) chose Figure 3 (BMI = 17.1) and explained: “because I like it, because I don’t want to be thin because I have little strength”. This desire to be stronger also shows the desire to defend oneself, for example: “I would like to look like this (pointing Figure 4, BMI = 21.03) because he is stronger like Doraemon cartoon. There’s one called giant and he’s very strong and I want to be like him. He’s a giant who beats” (boy, 4.6 years, BMI = 13.96).

Regarding endurance and speed, a 6.1-year-old girl (BMI = 25.57) chose Figure 1 (BMI = 13.03) as ideal and explained: “because she has a smaller belly and so I get less tired when running”. Similarly, a 5.6-year-old boy (BMI = 15.7) who also chose Figure 1 (BMI = 13.13) as ideal stated: “to run more, because my mother has told me that those who are thinner run faster”.

Theme 6: Body ideal. Within this theme are answers regarding the desire to have a different body size, the desire to grow up, and weight- and non-weight-related aspects of their bodies.

Desire for a body size. Some children wanted to be thinner or obese because of the meaning that these body sizes had for them. For example, many times, the children related the desire of a thin, obese, or medium body with other themes or subthemes such as nutrition, beauty ideals, the desire to grow up, or the desire to have better physical abilities, as aforementioned.

The mention of specific body size was more prevalent in females (12.6% vs. 7.5%). As children aged, they alluded more times to the desire for a body size: from 4 to 4.4 years, 5.1% of children mentioned body size, whereas at 6 years old, 21.7% did it.

Desire for weight-related aspects of their bodies. Nine children alluded to the size of legs, arms, and bellies (e.g., “because she has longer legs than mine”—girl, 4 years old, BMI = 13.52; “because he’s very cool and has long arms”—boy, 5.1 years old, BMI = 18.5; “because she has a flatter tummy”—girl, 5.8 years old, BMI = 14.2).

Desire for non-weight-related physical aspects of children’s bodies and other aspects. Although some children chose an ideal figure different from the perceived figure, they were not always unhappy with their body because sometimes, the reasons for selecting the figures had no relation to the body. For example, nine children chose the desired figures based on clothes (e.g., “I would like to look like this one because she has a short t-shirt that shows the belly”—girl, 5.6 years old, BMI = 13.47; “because I like his trousers”—boy, 6.2 years old, BMI = 17.12).

Desire to grow up. Twenty-two children longed to grow up in a similar manner at all ages, although the prevalence between 4.5 and 4.9 years was higher. To express their natural desire to grow up, some children used terms such as big and small, relating them to body weight and body size. For example, a boy aged 5.3 years (BMI = 13.6) responded: “I would like to look like this, the fat one (pointing Figure 4, BMI = 21.03), because I like it, I want to be large like him”.

Children also associated the desire to grow up with nutrition. For example, a girl aged 5 years (BMI = 17.9) stated that she drank too much milk to grow a lot. Likewise, a 4.8-year-old boy (BMI = 13.84) said: “because I eat a lot to become larger”.

## 4. Discussion

This study applied a mixed design to broaden the understanding of body image of children aged 4 to 6 years old, asking them about their perceived and ideal figure and the reasons why they chose these figures.

### 4.1. Accuracy in the Estimation of Body Size

Results suggest that children might have difficulties when attempting to differentiate their body size because we observed a high prevalence of inaccurate perception, which reinforces the literature [4,5,6,9,33] and is consistent with our first hypothesis. Furthermore, children’s body image was not stable because their perception was sometimes based on information obtained from specific nearby events or external evidence (e.g., if a child had recently received a comment on their body or had eaten a lot or a little the day before, that child could perceive their body size as different from reality). Thereby, their body image is arbitrary and changing [34] because the lack of conservation of self prevents them from having a fixed conceptualisation of themselves [35].

Body size perception varied by figure scale. With FF, both genders at all ages were more accurate in the perception of their body size, which may be because the profile scale provides less information on the body and could imply more biases since the belly is the most prominent feature and where body fat is very appreciable. Furthermore, according to Bauer et al. [36], the frontal view corresponds more with body perception in real life.

As we expected, the perception accuracy improved with age, although not in a progressive or linear way. This improvement was also observed by Cramer and Steinwert [3], whereas Tremblay et al. [9] found that age did not influence such perception. The improvements in body-size perception could be related to cognitive development but at these ages remain incipient. According to Palacios et al. [34], we observed that when the children reported self-descriptions, they tended to define themselves in absolute, concrete terms (e.g., I am tall) without qualifying these statements (e.g., I am tall for my age).

Body size perception tended to be more underestimated as children aged, especially in girls. These results, which may be due to the growing awareness of female body ideals, were consistent with those obtained by Meers et al. [5] and Tremblay et al. [9]. By contrast, Ra et al. [6] and Gardner [10] found that children had an overestimated body size perception, which has been interpreted as a reflection of their desire to grow up [14]. As for gender, Ambrosi-Randic and Tokuda [33] did not find significant differences between boys and girls, which is supported by our results. However, Holub [4] presented evidence that boys were more accurate than girls in the estimation of their real body size. According to this last author, such a finding could be related to the fact that young girls tend to report more body dissatisfaction than boys.

### 4.2. Are Young Children Dissatisfied with Their Bodies?

When children were asked to select their body ideal, we observed a desire for larger bodies than their perceived ones. However, as in the literature, we found that the desire for thinner bodies was more prevalent [6,37]. Such desire increased with age and was higher in girls [9,17,21,33], as we expected in our second hypothesis. This finding could be due to the link between self-esteem and physical attractiveness in women [38] and the internalisation of beauty ideals. In our study, girls were more likely to mention beauty ideals, although the prevalence was very low. In the case of males, we noticed the lack of the masculine beauty ideal. Explanations for this finding could be that the musculature was not represented in the figure scales [13]; at these ages, boys are not so committed to the adult beauty ideal; or boys confuse big with strong.

Although some children desired to be heavier or thinner, the majority showed satisfaction with their body as they selected the same figure as perceived and ideal, which supports our third hypothesis and is consistent with previous studies [17,21]. The reason for this finding could be because, at between 2 and 6 years of age, self-esteem tends to be idealised and give a positive bias [35]. By contrast, other authors have found high rates of body dissatisfaction, with percentages above 60% [5,8,9,18,33]. One possible explanation for this disparity in results could be the cultural differences in the study populations.

As in the literature [8,17], we found that the prevalence of body satisfaction was similar in all age groups. According to Tatangelo et al. [23], this finding may suggest that the differences by age emerge after the preschool stage. Regarding gender, our findings support the third hypothesis and largely agree with literature. Girls were more dissatisfied with their bodies than boys [9,16,17,21,33], possibly because of greater pressure than boys to achieve certain body ideals since early childhood [39]. By contrast, other researchers have not found gender differences in body dissatisfaction [8] and argue that these differences do not appear until adolescence [18].

Identifying body dissatisfaction among children at these ages is a concern because when children choose thinner ideal figures, their responses have tended to be systematically interpreted that, as with adults, they aspire to Western beauty ideals [9]. However, concurrent with previous research [16], our data show that although some children chose an ideal figure different from their perceived figure, they were not always dissatisfied with their bodies. Regarding this, Ricciardelli and McCabe [40] indicated that body dissatisfaction is determined by the value that the child places on having a certain body size and how this value can influence their life.

### 4.3. Reasons for Choosing Perceived and Ideal Figure

As Smolak [14] states, children can choose the perceived and ideal figures for different reasons that are not exclusively related to body size or weight, which was expected and observed in this study. This suggests that what children consider important might be inconsistent with the interpretations of adults [13,27] and that we cannot assume that they always judge body size when they are presented with a figural stimuli. Even when they selected a figure by body size, they sometimes did it for what a certain size represented for them. Among the mentioned reasons, we observed, for example, the desire to grow up, physical abilities, mother’s comments, and nutrition.

In our study, as in the literature [13,40], we found that children, especially younger children, reported a desire to grow up and tended to desire heavier bodies than the 6-year-old children. The desire to grow up stemmed from their ambition to be adults, have more power, imitate adults’ behaviours, do their own will, and decide freely [41].

In agreement with the findings of Slaughter and Ting [42], we found that the children also associated the desire to grow up with nutrition because some said they ate a lot to become much larger and grow up. We also observed that the children were aware that a certain body size or shape was linked to the amount and type of food, which had been found by Slaughter and Ting [42] and Rodgers et al. [43] in preschoolers.

Physical ability was an important theme reported often by children, which was also observed by Tatangelo and Ricciardelli [23] with preadolescents. Some children of our study associated thinness with speed and endurance and obesity with strength. Their responses showed some domains that integrate physical self-concept: physical strength, physical condition, and sport competence [44], demonstrating that physical aspects are relevant in how satisfied they are with their body and what it is able to do. Similarly, Palacios et al. [34] pointed out that at these ages, physical competence is a critical domain of self-esteem, and Birbeck and Drummond [13] argued that good physical abilities ensure that children are more accepted by their friends and gain prestige. In fact, Drummond [45] indicates that in childhood, the social hierarchy is based on sport and it seems especially true for boys. According to these authors, physical fitness is more important for boys because it enables them to demonstrate their masculinity. These findings are congruent with our results that found a higher number of boys than girls mentioned physical abilities.

Taking into account that physical abilities and sports are important for children’s body image, some authors have investigated what kind of sports are associated with a positive or negative body image. For example, Davison, Earnest and Birch [46] conducted a study with girls aged 5 and 7 years and found that those girls that participated in aesthetic sports (e.g., figure skating, gymnastics) reported higher weight concern than those who participated in non-aesthetic sports (e.g., soccer, basketball).

In this study, we also observed that several social factors influenced the children’s choices of their perceived and ideal figures. One such factor was body-weight stigma, which reflects other studies’ finding: preschoolers selected thin figures as friends or playmates, and they attributed more positive adjectives or made more favourable comments about them compared with overweight and obese figures [19,27]. Such stigmatisation is a risk factor for poor body image and can appear at 3 years [7], although according to Harriger [47], children are not usually fully committed to prejudices towards obesity before the age of five We observed weight stigma in our sample starting at 4 years of age.

Children’s responses also reflected the influence of their families. Parents contribute to the development of their children’s self-concept by continuously conveying information to them based their own beliefs, values, and attitudes towards bodies using verbal descriptive information (e.g., “You are a big girl”) [48]. Indeed, some experts have mentioned the impact of parents’ comments on their children’s body image [14,23]. McLaughlin et al. [24] found that almost a third of girls pointed out their mother’s comments about their bodies as a factor that affected their body satisfaction.

In our study, children alluded only to their mother or grandmother, suggesting that women are the adults who spend the most time raising them and thus become their greatest reference or influential figure. Such is this influence that mothers often transmit to their children their own body dissatisfaction and weight concerns [30]. We also found that the influence of mothers was greater among girls, and as Smolak [7] argued, this phenomenon occurs because girls are more likely than boys to talk about physical appearance with their mothers. The impact of the mothers was also reflected in pregnancy because some children considered that a large body was a consequence of pregnancy, as Rodgers et al. [43] observed, or they mentioned it because their mothers were or had been recently pregnant, which was also observed by Hayes and Tantleff-Dunn [17].

Regarding body parts, the belly was the most mentioned part by children, as was observed by Rodgers et al. [43]. This may be because the belly is a strong visual cue in body size judgements: Studies show that accuracy in body size discrimination is predicted by the prominence of the belly [49]. Legs were another frequently mentioned body part in children’s body size selections. Hayes and Tantleff-Dunn [17] found that some girls aged between 3 and 6 years wanted to have thinner legs; thus, they might focus more on this body part at these ages. The prevalence of the children’s mentions of the belly and legs could be because these two body parts cause greater dissatisfaction in women [20].

As previously mentioned, the choice of the ideal or perceived figure was not always based on body size or beauty ideals. In our study, many children pointed to clothing or body parts that provided little information on the body size of the figure (e.g., hands, nails, feet, fingers, neck). Regarding this, other authors have found that preschoolers were more concerned with their clothes or hair than with their body [17,22], which, according to McCabe et al. [22], may be because the comments that children receive are more oriented towards hair, clothing, or general appearance.

### 4.4. Cognitive and Verbal/Communication Limitations

We observed conceptual confusion of body dimensions. Because of the children’s cognitive and verbal limitations, they used the terms big or small to allude to height because some shorter children perceived themselves as smaller, or those taller as bigger or heavier, regardless of their weight status [50], and to allude to their age. Thus, in the preoperational stage (aged 2 to 7 years), things are what they appear to be, and children think they are older because they are taller. In addition, as in previous research [13,27], we observed that some children associated height with thinness, whereas other children associated height with obesity. As these authors argued, this finding may occur because children have little experience with optical illusions and would be influenced by the dimensions of the figures; however, an alternative explanation could be the centration, a characteristic of preoperational thinking that means children only focus on an isolated dimension or part of an object or stimulus [51].

Some children had difficulty assessing the body or body parts in terms of size and shape. In this sense, Dunphy-Lelii et al. [52] claimed that children’s judgements on the size of their arms, legs, and torso improve with age, but have not yet reached the ceiling at the age of 6 years. The mention of a concrete body size (e.g., thin, obese, medium) was more prevalent among girls; thus, girls seem to have more knowledge of body dimensions or a greater ability to make their body judgements in terms of size and shape. Furthermore, we found that at an older age, children referred more frequently to body size, which seems to demonstrate an increase in body awareness and greater vocabulary richness.

Young children’s limited ability to categorise also suggests underdeveloped cognitive abilities. Children tend to categorise based on the two extremes of the same dimension. For example, individuals or things are big or small, good or bad [53]. Children in our study, especially 4-year-olds, rarely mentioned the medium-sized figures instead tending to use the terms skinny and chubby.

## 5. Strengths, Limitations, and Prospectives

This mixed-design study of a large sample is a novel contribution to the literature on the evolution of preschoolers’ body image especially by using qualitative methods, which provide a broader insight into children’s body image. Another strength was the interobserver agreement between the authors. This study has limitations: some responses could not be interpreted because of the scarce vocabulary of the children. Regarding the stimuli used, figures’ body parts are not placed at the same distance and the changes in body size are not consistent, given that this instrument is composed of real bodies. Further research, through mixed designs, could longitudinally analyse body image to understand how it continues to develop in older children.

## 6. Conclusions

Our results suggest that at preschool ages, body image is unstable, and children’s cognitive limitations could explain the lack of a body ideal or an inaccurate perception of body size, especially among 4-year-olds. Many children are happy with their bodies and wanting a different body does not always reveal body dissatisfaction because the reason for choosing a figure is not necessarily related to body size or weight. Even selecting a figure by body size can symbolise the child’s desire to grow up, for pregnancy, for physical ability, or other aspects that result from the social influences to which they are exposed. Thus, data generated by quantitative research should be interpreted with caution because the usual conclusion has been that children who choose different ideal figures are bodily dissatisfied. However, as young children are often aware of societal beauty ideals that can reveal a concern for their body and show biases towards weight, programmes that encourage and maintain positive body image and acceptance of others among this age group are much needed.

## Figures and Tables

**Table 1:** Frequencies (%) of body-size perception and body ideal according to gender and age.

Groups	Underest.		AP		Overest.		DT		DS		DH	
	PF	FF	PF	FF	PF	FF	PF	FF	PF	FF	PF	FF
Total (*N* = 395)	58.6	39.3	20.4	32.2	21.1	28.5	13.6	16.8	65.2	69.8	21.2	13.4
4–4.4 years _♂_ (*n* = 51)	54	25.5	20	27.4	26	47.1	12	13.7	70	72.6	18	13.7
4–4.4 years _♀_ (*n* = 27)	42.3	51.8	26.9	22.2	30.8	26	11.5	18.5	61.5	72.5	27	16
4.5–4.9 years _♂_ (*n =* 55)	47.3	43.6	21.8	29.1	30.9	27.3	9.1	10.9	74.5	74.5	16.4	14.6
4.5–4.9 years _♀_ (*n* = 38)	56.7	42.1	8.1	28.9	35.2	28.9	29.7	18.4	40.5	57.9	29.8	23.7
5–5.4 years _♂_ (*n* = 40)	55	33.3	30	38.4	15	28.3	5	10.2	72.5	76.9	22.5	12.9
5–5.4 years _♀_ (*n* = 39)	68.4	33.3	23.7	41	7.9	25.7	13.2	23.1	52.6	59	34.2	27.8
5.5–5.9 years _♂_ (*n* = 46)	68.2	34.8	18.2	30.4	13.6	34.8	9.1	10.9	72.7	80.4	18.2	8.7
5.5–5.9 years _♀_ (*n* = 53)	61.5	43.4	17.3	33.9	21.2	22.7	21.1	20.7	63.4	64.1	15.5	15.2
6–6.4 years _♂_ (*n* = 21)	76.2	52.4	14.3	38.1	9.5	9.5	14.3	14.3	76.2	80.9	9.5	4.8
6–6.4 years _♀_ (*n* = 25)	64	48	24	36	12	16	12	36	64	60	24	4

Note. Underest., underestimation; AP, accurate perception; Overest., overestimation; DT, desire to be thinner; DS, desire to be the same (children choose the same figure as perceived and ideal); DH, desire to be heavier.

**Table 2:** Mean (SD) of real, perceived, and ideal BMI, discrepancy scores in body size perception, and body dissatisfaction according to gender and age.

Groups	Real BMI (r BMI)	Perceived BMI (p BMI)		Ideal BMI (i BMI)		Accuracy (p BMI–r BMI)		Satisfaction (i BMI-p BMI)	
		PF	FF	PF	FF	PF	FF	PF	FF
Total (*N* = 395)	15.6 (1.75)	14.86 (2.51)	15.57 (2.44)	15.22 (2.6)	15.42 (2.41)	−0.82 (2.95)	−0.11 (2.91)	0.36 (2.58)	−0.14 (2.1)
4–4.4 y _♂_ (*n* = 51)	15.28 (1.48)	14.76 (2.12)	16.58 (2.82)	15.27 (2.42)	16.55 (2.85)	−0.47 (2.91)	1.31 (3.28)	0.53 (2.29)	−0.04 (2.45)
4–4.4 y _♀_ (*n* = 27)	16.04 (1.56)	16.55 (3.48)	15.2 (2.46)	17.2 (3.58)	14.91 (2.33)	0.5 (3.44)	−0.84 (2.92)	0.65 (3.97)	−0.29 (2.64)
4.5–4.9 y _♂_ (*n* = 55)	15.67 (1.38)	15.74 (3.09)	15.59 (2.68)	16.2 (3.03)	15.69 (2.6)	−0.06 (3.39)	−0.08 (2.83)	0.46 (2.55)	0.09 (1.73)
4.5–4.9 y _♀_ (*n* = 38)	15.88 (1.61)	15.39 (2.87)	16.03 (2.97)	15.66 (2.76)	15.92 (2.64)	−0.42 (3.3)	0.15 (3.56)	0.27 (3.21)	−0.11 (2.86)
5–5.4 y _♂_ (*n* = 40)	15.24 (1.58)	14.69 (2.52)	15.7 (2.27)	15.37 (2.64)	15.77 (2.41)	−0.54 (2.67)	0.53 (2.14)	0.68 (3.02)	0.07 (1.8)
5–5.4 y _♀_ (*n* = 39)	16.97 (2.2)	14.06 (1.47)	15.31 (1.99)	14.61 (1.8)	15.26 (2.37)	−1.86 (2.6)	−0.66 (2.87)	0.55 (1.96)	−0.05 (1.64)
5.5–5.9 y _♂_ (*n* = 46)	15.77 (1.7)	14.40 (1.91)	15.66 (1.86)	14.72 (2.37)	15.66 (2.2)	−1.43 (2.73)	−0.11 (2.52)	0.32 (2.59)	−0.001 (1.5)
5.5–5.9 y _♀_ (*n* = 53)	15.61 (1.91)	14.37 (2)	14.87 (2.09)	14.24 (1.87)	14.59 (1.71)	−1.28 (2.6)	−0.74 (2.86)	−0.12 (1.75)	−0.27 (2.2)
6–6.4 y _♂_ (*n* = 21)	16.07 (1.51)	14.48 (2.51)	15.35 (2.43)	14.24 (1.64)	15.11 (2.06)	−1.58 (2.69)	−0.71 (2.57)	−0.23 (1.64)	−0.23 (1.26)
6–6.4 y _♀_ (*n* = 25)	15.85 (2.48)	14.16 (1.96)	14.9 (2.28)	14.57 (2.06)	13.67 (0.96)	−1.69 (2.03)	−0.95 (2.38)	0.4 (2.77)	−1.22 (2.43)

**Table 3:** Description of themes and subthemes. Frequencies and percentages of themes according to age group and gender, including responses from perceived and ideal bodies.

Themes	Subthemes	Description	Children (*N*)	A%	B%	C%	D%	E%	Gender%
1. Body-size perception									
	1.1. Body size	Children perceive themselves as thin, obese, or medium (i.e., neither too thin nor too obese) or indicate that the size of their body is the same as that of the chosen perceived figure (i.e., the same size as me).	Thinness: 73; obesity: 11; medium-sized: 16; body size: 22 Total: 122	15.4	21.5	41.8	36.4	41.3	_♂_ = 29.6 _♀_ = 31.3
	1.2. Weight-related aspects of their bodies	Children mention body parts that provide information on body size (e.g., thin legs, long arms, thin belly, buttocks).	Belly: 39; legs: 9; arms: 5; buttocks: 2 Total: 55	7.7	15	16.4	14.1	10.9	_♂_ = 12.7 _♀_ = 13.7
	1.3. Non-weight related physical aspects of children’s bodies and other aspects	Children mention body parts that provide little or no information on body size (e.g., feet, hands, neck, fingers, nails, navel, and skin) and other aspects (e.g., age, clothes, and body position).	Skin: 5; age: 7; clothes: 38; body position: 10; feet: 25; hands: 21; nails: 5; navel: 16; neck: 3; fingers: 5; bones: 1 Total: 136	25.6	25.8	32.9	27.3	10.9	_♂_ = 27.7 _♀_ = 23.6
2. Conceptual confusion of dimensions		Children confuse some terms referring to dimensions (i.e., thin, obese, large, small, tall, short, long, old, or big).	40	14.1	10.7	8.4	9.1	8.7	_♂_ = 8.9 _♀_ = 11.5
3. Influence of the social context									
	3.1. Weight stigmatisation	Children make fun of or speak negatively of overweight or obese figures.	5	0	2.5	1.3	1	2.2	_♂_ = 1.9 _♀_ = 0.55
	3.2. Beauty ideal	Children mention social beauty ideals related to thinness.	4	0	1.1	1.3	0	4.3	_♂_ = 0.47 _♀_ = 1.65
	3.3. Nutrition	Children relate body size to the amount and type of food.	15	3.8	8.6	1.3	2	2.2	_♂_ = 5.6 _♀_ = 1.6
4. Mother’s influence		Children allude to their mother’s comments about the body. It also included answers about pregnancy.	13	1.3	2.1	2.5	5	6.5	_♂_ = 0.94 _♀_ = 6.04
5. Physical abilities		Children mention basic physical abilities such as strength, endurance, or speed and associate them with a certain body size.	10	0	3	3.4	2.5	4	_♂_ = 3.75 _♀_ = 1.1
6. Body ideal									
	6.1. Desire for a body size	Children want to be thinner, larger, or medium-sized (i.e., neither too thin nor too obese) or indicate that they want to have the same body size as the figure chosen as ideal.	Thinness: 19; obesity: 12, medium-sized: 6; body size: 2 Total: 39	5.1	7.5	10.1	10.1	21.7	_♂_ = 7.5 _♀_ = 12.6
	6.2. Desire for weight-related aspects of their bodies	Same description as in subtheme 1.2	Belly: 5; legs: 2; arms: 2 Total: 9	2.6	1.1	3.8	1	4.3	_♂_ = 1.4 _♀_ = 2.75
	6.3. Desire for non-weight-related physical aspects of children’s bodies and other aspects	Same description as in subtheme 1.3	Skin: 3; age: 1; fingers: 1; hands: 7; feet: 6; body position: 5; navel: 5; clothes: 9 Total: 37	5.1	8.6	8.9	12.1	4.3	_♂_ = 6.6 _♀_ = 10.4
	6.4. Desire to grow up	Children desire to grow up and be older.	22	2.6	9.7	5	5	4.3	_♂_ = 3.75 _♀_ = 7.65

Note: Age groups: A, 4 to 4.4 years; B, 4.5 to 4.9 years; C, 5 to 5.4 years; D, 5.5 to 5.9 years; E, 6 to 6.4 years. Themes 2, 3, 4, and 5 include responses from both perceived and ideal bodies. Theme 1 only comprises responses from perceived bodies and theme 6 from ideal bodies.
